# Arthroscopic Treatment of Scapholunate Instability: A Stage- and Ligament-Based Narrative Review

**DOI:** 10.3390/medicina62091706

**Published:** 2026-09-05

**Authors:** Youn-Tae Roh, Dong In Seo, Il-Jung Park

**Affiliations:** 1 Department of Orthopaedic Surgery, Seoul St. Mary’s Hospital, College of Medicine, The Catholic University of Korea, Seoul 06591, Republic of Korea; ryt003@gmail.com (Y.-T.R.); seodong159@naver.com (D.I.S.); 2 Department of Orthopaedic Surgery, Bucheon St. Mary’s Hospital, College of Medicine, The Catholic University of Korea, Seoul 06591, Republic of Korea

**Keywords:** scapholunate ligament, carpal instability, wrist arthroscopy, arthroscopy, ligament reconstruction

## Abstract

Scapholunate (SL) instability is the most common form of carpal instability, and if untreated, it may progress through dorsal intercalated segment instability to scapholunate advanced collapse. Contemporary understanding emphasizes a dual stabilizing system in which the SL interosseous ligament (SLIL) acts as the intrinsic primary restraint, reinforced by extrinsic “critical” secondary stabilizers; isolated SLIL division does not by itself produce static malalignment until these secondary restraints are also compromised. Against this background, wrist arthroscopy has become a widely used reference standard for diagnosis and grading, allowing for graded assessment through the Geissler and the more granular European Wrist Arthroscopy Society (EWAS) classifications, while dry arthroscopy has reduced fluid-related morbidity and facilitated combined arthroscopic and mini-open procedures. Treatment has correspondingly shifted from open reconstruction toward arthroscopic and arthroscopic-assisted repair, capsulodesis, and ligamentoplasty, which are intended to preserve native capsuloligamentous structures and may offer potential advantages in limiting capsular disruption and postoperative stiffness. This narrative review synthesizes the literature identified through a selective search of PubMed/MEDLINE, Scopus, Web of Science, and Google Scholar (final search 13 June 2026). The evidence consists mainly of small observational series and technique reports. The review stages the injury by arthroscopic grade (EWAS/Geissler) and organizing techniques by the target ligament(s), from partial and dynamic injuries through complete, reducible, and advanced deformities. We align each technique with the corresponding pattern of intrinsic and extrinsic ligament injury to clarify indications, summarize reported outcomes, and propose a stage-based conceptual treatment framework rather than a validated, evidence-based guideline. Current evidence derives largely from small, heterogeneous case series with variable classifications and outcome measures, underscoring the need for standardized staging and comparative studies.

## 1. Introduction

Scapholunate (SL) instability is the most common form of carpal instability and may progress, if left untreated, to scapholunate advanced collapse (SLAC) [1,2]. Its stability depends not only on the intrinsic SL interosseous ligament (SLIL) but also on a network of extrinsic critical stabilizers, including the dorsal intercarpal (DIC) ligament, the dorsal radiocarpal (DRC) ligament, the dorsal capsulo-scapholunate septum (DCSS), and the long radiolunate (LRL) ligament, whose proposed role in maintaining the synchronous proximal carpal row kinematics has been described in recent biomechanical and anatomical work [1,2,3,4,5].

Historically, the management of chronic or severe SL instability has relied on a variety of open surgical interventions [1,6]. Although these open reconstructions can restore carpal alignment, they typically require extensive dorsal approaches that inevitably disrupt the critical extrinsic stabilizers and the joint capsule, and they frequently result in postoperative stiffness, reduced range of motion, and unpredictable long-term outcomes [1,6,7]. Over the past two decades, wrist arthroscopy has transformed the diagnosis and treatment of SL injuries [1,8]. Initially employed for debridement and electrothermal shrinkage in partial, stable tears (Geissler grades I–II) [9,10], arthroscopic techniques have evolved rapidly and now encompass sophisticated, minimally invasive repairs and reconstructions for high-grade dynamic and static instability. Innovations such as the all-inside volar SL suture [11], the arthroscopic dorsal capsuloligamentous repair (ADCLR) [12] and its wide variant (WADCLR) [13], and all-dorsal arthroscopic ligamentoplasty (ADAL) [14,15] demonstrate that multiplanar stabilization may be achievable through tissue-preserving arthroscopic approaches, while potentially limiting disruption of the native capsuloligamentous envelope.

Despite the rapid proliferation of these advanced techniques, significant knowledge gaps remain. A recent systematic review and meta-analysis highlighted the lack of high-quality randomized controlled trials (RCTs) demonstrating the clinical superiority of wrist arthroscopy over open surgery or nonoperative care [16]. Moreover, surgical indications in the literature are fragmented across different classification systems, notably the Geissler and European Wrist Arthroscopy Society (EWAS) gradings [17,18]. No unified framework yet integrates the current biomechanical understanding of the critical extrinsic stabilizers with modern arthroscopic staging. The unresolved clinical problem is therefore the absence of a clearly defined relationship between the arthroscopic findings, the specific stabilising structures that are injured, and the selection of an appropriate arthroscopic procedure [1,2].

Therefore, the objective of this narrative review is to synthesize the current literature on the arthroscopic management of pre-arthritic SL instability. By evaluating the anatomical targets, surgical techniques, and clinical outcomes of recent advances, we aim to propose a pragmatic, stage- and ligament-based conceptual framework for treatment selection [19]. This framework seeks to match specific arthroscopic interventions to distinct EWAS and Geissler injury stages, thereby facilitating targeted, tissue-specific surgical decision-making and highlighting avenues for future high-level research.

## 2. Research Strategy

This narrative review was conducted to synthesize the current literature on arthroscopic and arthroscopic-assisted treatment of pre-arthritic scapholunate (SL) instability, with particular emphasis on the relationship between arthroscopic stage, injured ligamentous structures, surgical technique, and reported clinical outcomes.

A literature search was performed in PubMed/MEDLINE, Scopus, Web of Science, and Google Scholar; the final search was conducted on 13 June 2026 and included publications available up to that date. The search strategy combined Medical Subject Headings (MeSH) and free-text terms related to scapholunate instability and arthroscopic treatment. The core search concepts included “scapholunate,” “scapholunate dissociation,” “scapholunate instability,” “scapholunate interosseous ligament,” and “SLIL,” combined with terms related to arthroscopy and operative treatment, including arthroscop*, capsulodesis, capsuloligamentous repair, ligamentoplasty, suture anchor, thermal shrinkage, EWAS, and Geissler. Additional technique-specific terms, including ADCLR, WADCLR, ADAL, RADICL, MIRLIN, internal brace, dorsal scaphotriquetral ligamentoplasty, and 360-degree reconstruction, were also considered when identifying relevant literature. Because this was a selective narrative search rather than a protocol-driven systematic review, database-specific reproducible search strings were not prospectively archived; the search concepts actually applied are therefore described here rather than reconstructed retrospectively as database-specific strategies.

Studies were considered eligible when they addressed the diagnosis, classification, biomechanics, or arthroscopic/arthroscopic-assisted treatment of scapholunate instability and provided clinical, biomechanical, anatomical, or technical information relevant to the objectives of this review. Clinical series, retrospective and prospective observational studies, technique reports, biomechanical or anatomical studies, and selected case reports were considered when they contributed substantially to the understanding of a specific arthroscopic technique or injury pattern. Seminal publications describing established classifications or surgical techniques were retained irrespective of publication year.

Studies were excluded when they were unrelated to scapholunate instability or arthroscopic treatment, focused exclusively on established degenerative SLAC wrist without relevance to pre-arthritic instability, or did not provide information relevant to the review objectives. No language filter was applied at the search stage; all sources ultimately drawn upon were published in English. When multiple publications described the same technique or patient cohort, we retained the reports that provided the most relevant clinical or technical information for the purposes of this review, while also retaining earlier publications when they provided the original technical description or important historical context.

Study identification and selection were performed by review of titles and abstracts followed by assessment of potentially relevant full-text articles; because this was a narrative review, formal independent duplicate screening was not performed. Study selection was purposive and based on relevance to the review question, methodological contribution, clinical importance, and availability of contemporary evidence. Reference lists of key articles and reviews were additionally screened to identify relevant publications that might not have been retrieved by the electronic search, including selected book chapters and technical descriptions. Aggregate record counts were not prospectively logged, and numerical screening counts have therefore not been reconstructed; the review cites 30 sources in total, of which 15 were clinical or technique-related sources.

The literature was synthesized narratively rather than quantitatively. Particular attention was given to the arthroscopic EWAS stage, the approximate relationship with Geissler grading, chronicity, ligament quality and repairability, reducibility, radiographic deformity, cartilage status, and the specific intrinsic or extrinsic ligamentous structures involved. Clinical outcomes were interpreted in the context of study design, sample size, follow-up duration, and the presence or absence of comparative controls. No formal meta-analysis or quantitative risk-of-bias assessment was performed.

**Figure 1:**
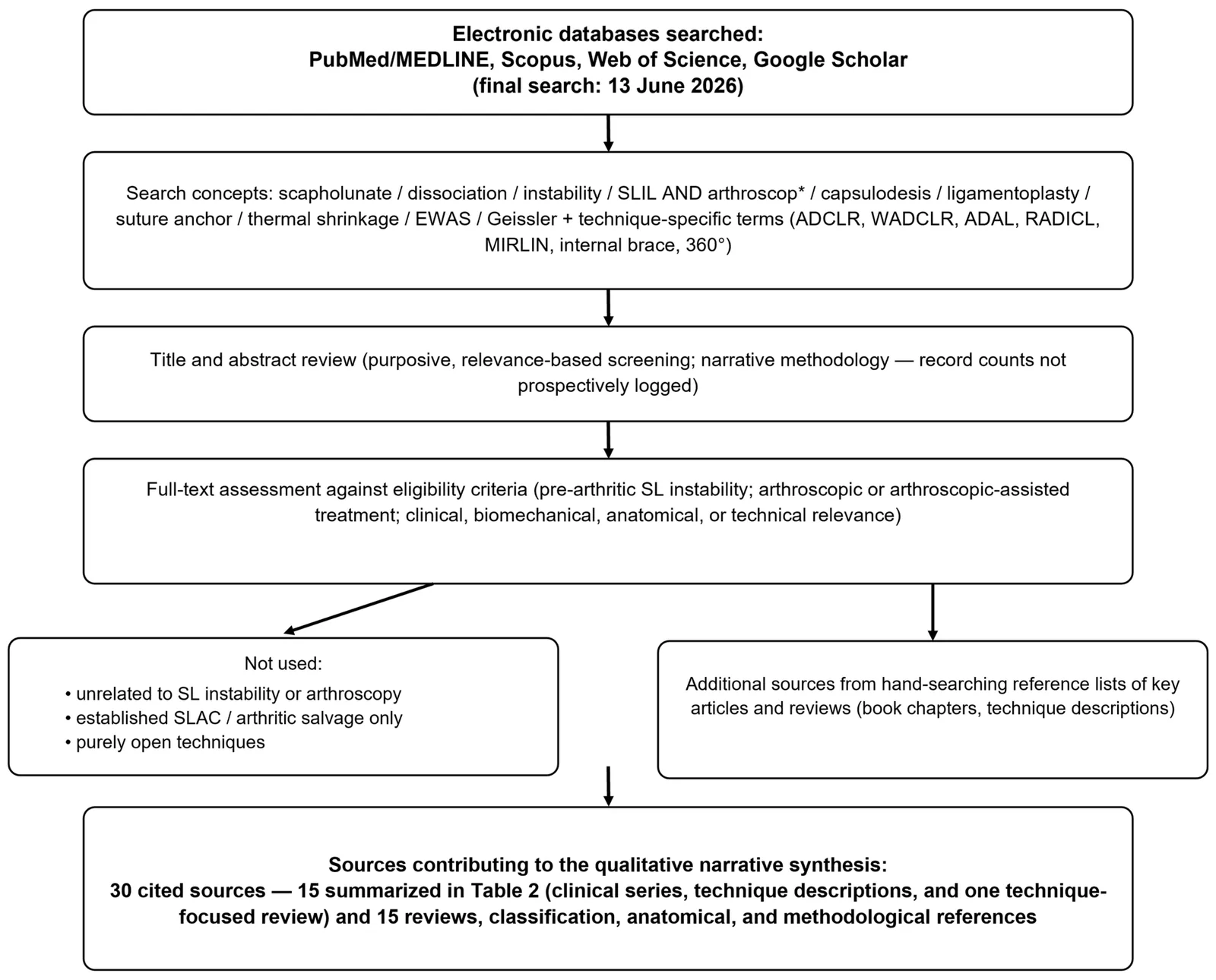
Flow diagram of the literature identification and selection process for this narrative review. Records identified in PubMed/MEDLINE, Scopus, Web of Science, and Google Scholar (final search, 13 June 2026) were screened by title and abstract, assessed in full text for relevance, and supplemented by hand-searching of the reference lists of key articles and reviews. Because this is a selective narrative rather than a systematic review, sources were selected purposively according to relevance to the review question and their methodological or clinical contribution, and aggregate record counts were not prospectively logged; the synthesis ultimately drew on 30 cited sources, of which 15 were clinical or tech-nique-related. The asterisk in ‘arthroscop*’ denotes a truncation (wildcard) operator used in the database searches.

Because the available literature is heterogeneous and is dominated by small observational series and technical reports, the treatment framework presented in this review is intended as a pragmatic, opinion-based conceptual framework rather than a validated clinical guideline. The literature identification and selection process is summarized in Figure 1.

## 3. Main Body

### 3.1. Anatomy and Pathomechanics Relevant to Arthroscopic Treatment

**Figure 2:**
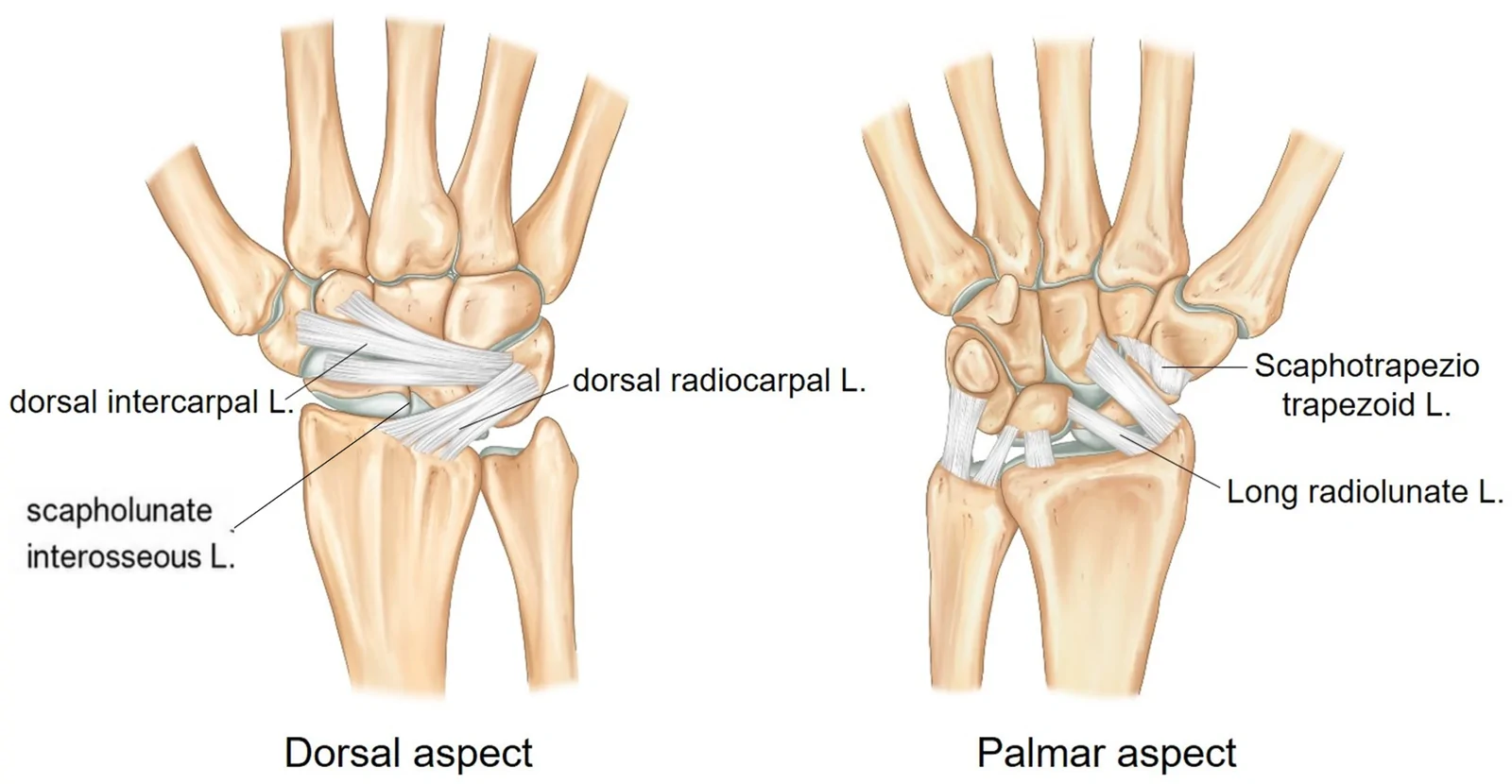
Dorsal and palmar ligamentous anatomy of the wrist, illustrating the extrinsic “critical” secondary stabilizers of the scapholunate joint. The labeled structures are the dorsal intercarpal ligament (DIC), the dorsal radiocarpal ligament (DRC), the scaphotrapeziotrapezoid ligament (STT), and the long radiolunate ligament (LRL); the position of the scapholunate interosseous ligament (SLIL), the intrinsic primary stabilizer, is indicated at the scapholunate interval. The dorsal capsulo-scapholunate septum (DCSS) lies deep to the dorsal capsule between the DIC and the dorsal SLIL and is therefore not shown as a separate structure at this scale.

Stability of the SL joint rests on a dual ligamentous system in which the SL interosseous ligament (SLIL) acts as the intrinsic primary stabilizer, reinforced by a network of extrinsic stabilizers [1,3,4]. The C-shaped SLIL comprises three regions: a thick, strong dorsal region that is the principal restraint to distraction, translation, and rotation; an obliquely oriented volar region governing sagittal rotation; and a fibrocartilaginous proximal membranous region of little mechanical importance [1,3,4]. Recent anatomical and biomechanical work has emphasized that these extrinsic capsuloligamentous structures, namely the dorsal intercarpal (DIC) ligament, the dorsal radiocarpal (DRC) ligament, the long radiolunate (LRL) ligament, the scaphotrapeziotrapezoid (STT) ligament, and the dorsal capsulo-scapholunate septum (DCSS), are not merely secondary restraints but essential “critical stabilizers” that govern synchronous carpal motion (Figure 2) [3,5]. The DIC and DRC in particular insert firmly onto the cartilage-free dorsal “bare area” of the lunate, strongly supporting the proximal carpal row [3]. Together, this intrinsic–extrinsic system enables the scaphoid and lunate, which lack direct tendinous attachments, to move as a synchronized intercalated segment between the proximal and distal carpal rows [3,4].

Pathomechanically, isolated rupture of the SLIL alone does not immediately produce static deformity such as radiographic SL gapping or DISI [1]. Frank SL diastasis and DISI emerge only when rupture or attenuation of the critical extrinsic ligaments, namely the DIC, DRC, and LRL, accompanies the SLIL injury; additional failure of one or more of these important secondary stabilizers, beyond the SLIL itself, is generally associated with the development of static malalignment, although the specific combination of ligament injuries producing static deformity varies among experimental models and clinical injuries [3,8]. This accounts for the clinical spectrum, in which the injury progresses from dynamic instability apparent only under load to fixed static malalignment as successive stabilizers fail [1,8]. Once such multiligamentous failure occurs, the scaphoid flexes and pronates while the lunate extends, producing the abnormal kinematics and progressive carpal collapse that, untreated, culminate in scapholunate advanced collapse [5].

These principles provide a rationale for arthroscopic treatment: because the conventional dorsal ligament-splitting approach may disrupt the DIC and DRC insertions that contribute to carpal stability, and may itself induce iatrogenic DISI, capsule- and insertion-preserving arthroscopic techniques may offer a tissue-preserving alternative, potentially limiting disruption of the secondary stabilizers and the native capsuloligamentous structures. Any effect on capsular vascularity and proprioceptive function is a theoretical rather than an established benefit, as it has not been demonstrated in direct comparative clinical studies [1,3,7].

### 3.2. Clinical Examination, Imaging, and Arthroscopic Diagnosis: Classification and the Enabling Role of Dry Arthroscopy

Diagnosis begins with clinical examination. Patients typically present with dorsoradial wrist pain aggravated by loading, and tenderness is localized over the SL interval; provocative maneuvers such as the Watson scaphoid shift test can reproduce the patient’s pain and a clunk, raising suspicion of SL instability, although the sensitivity and specificity of provocative testing are limited [1,3,8].

Imaging provides complementary, noninvasive staging information [8]. On static radiographs, SL gapping, an increased SL angle (over 70°), lunate extension (over 15°), a cortical ring sign from scaphoid flexion, and dorsal scaphoid translation suggest static instability [3,8]. When static films are normal, stress views (clenched-fist, twist, and pencil-grip radiographs) may unmask dynamic widening, and dynamic fluoroscopy, which follows the carpus through motion in real time, has a reported sensitivity of 90% and specificity of 97% for SL instability [8]. Among cross-sectional techniques, CT arthrography also depicts cartilage status and has been reported to have a sensitivity of 94% and a specificity of 86% for SLIL tears when arthroscopy was used as the reference standard, while a meta-analysis comparing magnetic resonance protocols reported a sensitivity of 82% and a specificity of 93% for MR arthrography, with 3-T MRI showing the highest specificity within that comparison [8]. These figures derive from different primary studies with differing lesion definitions and reference standards and are therefore not directly comparable across modalities [8]. Imaging alone, however, cannot reliably distinguish partial from complete tears or fully assess the extrinsic stabilizers [8].

For definitive assessment, wrist arthroscopy provides the most direct reference standard for the assessment and grading of scapholunate instability, particularly in partial and dynamic injuries. It is, however, invasive, and arthroscopic grading may be subject to interobserver variability; arthroscopy therefore complements rather than replaces clinical and imaging evaluation [1,4,8]. From the radiocarpal joint, the dorsal, proximal, and volar regions of the SLIL are inspected directly, while dynamic testing is performed from the midcarpal portals: a probe introduced into the SL interval assesses widening, laxity, and reducibility under direct vision, forming the basis of the EWAS grading [18]. The insertions of the DIC and DRC onto the dorsal lunate and the DCSS can also be visualized from the 6R, 1–2, and midcarpal portals, where a bare area of the dorsal lunate is considered a characteristic finding suggestive of extrinsic-ligament avulsion [20]. Not all extrinsic stabilizers, however, are completely assessable through routine portals; the volar and ulnar-sided restraints in particular are only partially visualized, so arthroscopic staging is interpreted together with the clinical and imaging findings [1,8].

Classification has evolved through successive arthroscopic schemes. Geissler graded instability by progressive incongruity, from interosseous attenuation seen radiocarpally, to a midcarpal step-off with probe passage, to passage of the arthroscope across the interval [4,17]. In 2013, Messina et al. introduced the European Wrist Arthroscopy Society (EWAS) classification, assessed from the midcarpal radial portal, which spans stage I (an intact but lax ligament that the probe cannot enter), stage II (probe-tip entry without widening, stable), stage III (dynamic widening, subdivided into IIIA volar, IIIB dorsal, and IIIC complete but reducible), stage IV (a complete tear allowing passage of the arthroscope from the midcarpal to the radiocarpal joint, with radiographs still normal), and stage V (a complete tear with established radiographic changes such as SL diastasis or DISI) [18,19]. In the original 2013 description, stage II is a single category corresponding to a lesion of the membranous portion of the SLIL, and the A–C subdivisions are specified only for stage III. In the expanded form of the classification used subsequently in clinical practice, stage II is itself subdivided by the site of probe passage into IIA (volar), IIB (dorsal), and IIC (complete), in a manner analogous to the stage III subdivisions [12]. Because the present review organises treatment by the injured ligament, Table 1 records both the original stage II category and this expanded subdivision, which preserves information on the location and extent of the lesion. By correlating these arthroscopic findings with the underlying anatomopathology, the EWAS system supports tissue-specific treatment selection, and it has been applied in this way in recent clinical practice [19]. The Andersson–Garcia-Elias classification adds a complementary descriptor of the dorsal SLIL injury pattern, avulsion versus mid-substance, that informs repairability [1]. Correspondence between the EWAS and Geissler systems, however, remains approximate rather than standardized (Table 1). The two schemes capture different information: Geissler grading reflects the degree of arthroscopically demonstrable widening and instability, whereas the EWAS system also encodes the anatomical location and extent of the SLIL lesion and its behaviour on dynamic testing. In particular, the IIA–IIC subdivisions record whether probe passage occurs volarly, dorsally, or across the whole interval, a distinction that a Geissler grade does not convey. EWAS staging should therefore be regarded as complementary to, rather than interchangeable with, Geissler grading when characterising SLIL injury.

Finally, the utility of arthroscopic diagnosis and treatment is maximized by the dry arthroscopy technique, performed under traction alone without fluid infusion. With this approach, a minimally invasive mini-open reconstruction can be performed immediately and seamlessly after arthroscopic diagnosis, preserving the soft-tissue planes required for such combined (“hybrid”) procedures [21]. The arthroscopic and arthroscopic-assisted techniques discussed in Section 3.3, Section 3.4 and Section 3.5 are summarized, together with their study designs, levels of evidence, and reported complications, in Table 2.

### 3.3. EWAS I–II (IIA-C; Approx. Geissler I–II): Occult and Partial (Stable) Injuries

EWAS I–II, approximately corresponding to Geissler I–II, denotes an early stage in which an occult or partial tear of the scapholunate (SL) ligament complex has occurred but the overall mechanical stability of the joint is preserved [1]. Patients typically report persistent dorsoradial wrist pain on forceful gripping or weight-bearing, a very common cause of chronic wrist pain that responds poorly to nonoperative care [10]. Arthroscopically, EWAS I (approximately corresponding to Geissler I) is the earliest state, showing only hemorrhage or attenuation of the SLIL when viewed from the radiocarpal joint, with no midcarpal incongruency and no passage of the probe across the interval [17,18]. The slightly more advanced EWAS II (approximately corresponding to Geissler II) reflects injury largely confined to the proximal membranous portion of the ligament; from the midcarpal joint a slight step-off or incongruency is seen and the tip of the probe can be introduced into the interval, but the gap does not widen on dynamic testing and the lesion is therefore still classified as stable [4,18]. In the expanded form of the classification, the site of probe passage further distinguishes IIA (volar), IIB (dorsal), and IIC (complete), which identifies the injured limb of the ligament even though the joint remains stable [12].

For these stage I–II partial injuries, initial treatment favors conservative management over immediate surgery: rest with splint immobilization combined with proprioceptive physiotherapy and re-education of the SL-friendly musculature, such as the flexor carpi radialis (FCR) [1]. When mechanical pain and symptoms persist despite an adequate trial of conservative management, minimally invasive arthroscopic surgery may be considered [27]. The most widely performed interventions at this stage are arthroscopic debridement and electrothermal collagen shrinkage using a bipolar radiofrequency (RF) probe (Table 2) [4,10].

Debridement alone, which removes the frayed ligament edges and associated synovitis, was associated with satisfactory pain relief in approximately 85% of patients with partial tears in one retrospective series [9]. Electrothermal shrinkage has been used with the intent of reducing laxity and improving ligament tension: under arthroscopic control, controlled radiofrequency heat denatures and shortens the collagen within the stretched, attenuated ligament, causing it to contract. The durability of this effect and its ability to provide sustained mechanical stability remain uncertain, recurrent laxity may occur, and thermal energy carries a potential risk of injury to articular cartilage and adjacent neural structures. Given the limited and relatively old clinical evidence, this technique may be considered only cautiously [10]. To limit thermal injury to cartilage, the RF probe is applied intermittently and judiciously. When electrothermal shrinkage is performed, fluid irrigation is deliberately maintained to provide cooling and to reduce the risk of thermal injury; this differs from the dry technique described for selected purely arthroscopic procedures, and the two approaches are therefore complementary rather than contradictory [10,21]. In selected patients with acute Geissler II injuries in whom some instability is suspected, arthroscopic reduction and percutaneous Kirschner-wire pinning, an approach pioneered by Whipple, may be combined with debridement [4,28]. A systematic review of partial SLIL injuries, which pooled predominantly Level IV case series together with one Level III case–control study, reported that these arthroscopic interventions were associated with improvements in pain, motion, grip strength, and the radiographic scapholunate gap, with low complication rates. That review also included a small untreated cohort; this cohort was drawn from a separate study rather than serving as a concurrent control group, so the comparison between treated and untreated wrists is indirect and cannot establish treatment efficacy relative to no treatment. Comparative data therefore remain insufficient to define the optimal technique [28].

### 3.4. EWAS III (IIIA–C; Approx. Geissler III): Partial to Complete, Reducible Injuries

EWAS stage III, which corresponds approximately to Geissler III, encompasses a spectrum of injury from partial to complete tears of the SL ligament complex, but the injuries share one essential feature: the carpal malalignment remains reducible [1,18]. Patients may show normal static radiographs yet demonstrate dynamic instability, with clear widening of the SL interval under dynamic stress such as gripping or loading [4,8]. This is the domain in which arthroscopic repair is most actively applied, and treatment is increasingly matched to the specific portion of the SLIL and to the secondary stabilizers involved [1,4].

EWAS IIIA denotes anterior laxity arising from a partial tear of the volar SL ligament [18]. For this region, historically difficult to reach by open approaches without dividing the volar extrinsic ligaments, del Piñal et al. described an innovative all-inside volar repair that, using only a Tuohy needle and an absorbable suture, reefs the volar capsule together with the long radiolunate (LRL) ligament to achieve minimally invasive closure of the anterior interval; the authors described preliminary experience in four patients with insufficient follow-up for outcome analysis [11].

EWAS IIIB shows posterior laxity from partial injury of the dorsal SL ligament and the dorsal intercarpal (DIC) ligament [18]; here the dorsal capsuloligamentous structures are the target. Mathoulin’s arthroscopic dorsal capsuloligamentous repair (ADCLR) reattaches the dorsal capsulo-scapholunate septum (DCSS) and the SLIL remnant to the dorsal capsule without open capsulotomy, restoring the native anatomic tension; in a retrospective series of 221 patients, satisfaction was reported in 215 of 221 patients (reported as 95.7%), the DASH improved from 47.04 to 9.4, and a DISI deformity persisted in 43 patients (19%) [12]. Other dorsal techniques instead target the extrinsic secondary stabilizers directly. For dynamic instability, Im et al. described an arthroscopic dorsal scaphotriquetral ligamentoplasty linking the scaphoid and triquetrum with SutureTape and a 2.5 mm PushLock anchor; in thirteen isolated SL injuries the visual analog scale fell from 7.9 to 1.4 and grip rose from 27.4 to 37.3 kg, with proximal-row motion preserved [26]. The RADICL technique, described by Williams et al., reattaches the avulsed dorsal intercarpal and dorsal radiocarpal ligaments to the dorsal lunate bare area; in a small institutional case series of five patients (three female, two male; average age 40 years) reported within a book chapter, with outcomes at six months, pain scores roughly halved (49.4 to 23.8), QuickDASH and PRWE improved, and grip increased from 19.6 to 33.6 kg [20].

EWAS IIIC represents a complete but reducible tear in which the volar, dorsal, and proximal portions of the SLIL are all disrupted, often with complete failure of one extrinsic ligament (e.g., the DIC or the RSC/LRL). The optimal extent of repair is debated. Chambers et al. argued that, given this wide extent, both limbs should be addressed, arthroscopically shuttling the volar and dorsal capsuloligamentous tissues and securing them with a modified Nice knot; in eight patients with predynamic injuries, function, motion, and pain relief were excellent with minimal radiographic change [19]. In contrast, Bain and Amarasooriya conceptualize SL instability primarily as a failure of the dorsal scapholunate complex, particularly its scaphoid attachment, and advocate a dorsal-focused biological repair, implying that a well-executed dorsal reconstruction may restore stability without a separate volar procedure [5]. In acute and subacute tears, Lee et al. combined arthroscopic suture-anchor repair of the SLIL with dorsal capsulodesis incorporating the DRC, achieving good-to-excellent Modified Mayo Wrist Scores in 94.7% with a single recurrence [23]. When the remaining tissue is judged insufficient for repair, tendon-graft reconstruction may be required instead (Section 3.5).

Across these techniques, capsule- and insertion-preserving repair is intended to minimize capsular disruption and may potentially reduce postoperative stiffness; reported series describe improvements in pain, grip, and motion, and the modular options can be combined according to which ligaments are injured, although direct comparative evidence is lacking [4,27]. Nonetheless, the evidence remains heterogeneous and largely uncontrolled, the radiographic gap is not always normalized, and no single technique has been shown to be superior in comparative studies [27].

### 3.5. EWAS IV–V (Beyond Geissler IV): Complete, Reducible Injuries with or Without Radiographic Deformity

When instability progresses to a complete tear with an arthroscopically demonstrable SL gap and drive-through sign but still-normal radiographs (EWAS IV) or to a complete tear with established radiographic deformity such as SL diastasis or DISI (EWAS V), a severity beyond the Geissler grades, which end at IV, simple repair becomes insufficient, and treatment shifts toward wide capsuloligamentous repair or formal ligamentoplasty, provided the carpus remains reducible and the cartilage is preserved [1,13].

Recent advances have extended the limits of minimally invasive surgery for these high-grade injuries. For patients with insufficient ligament remnant for direct repair (a “bald” scaphoid) or with severe instability, de Villeneuve Bargemon et al. described the wide arthroscopic dorsal capsuloligamentous repair (WADCLR), which secures stability through aggressive debridement, extracapsular knots, and mini-anchors. In their prospective series of 112 patients spanning EWAS stages 3 to 5, they reattached the interosseous system to the extrinsic ligaments and recreated the dorsal capsulo-scapholunate septum, using an anchor where no stump remained, achieving normalization of radiographic parameters and improvement in all functional domains except flexion, with a single complication [13]. Acute, reducible stage-IV tears may still be amenable to the suture-anchor repair described in Section 3.4 [23].

When the ligament is irreparable, arthroscopic reconstruction aims to restore the SLIL with a tendon graft. Corella et al. reconstructed both the dorsal and volar limbs with a flexor carpi radialis graft passed through scaphoid and lunate tunnels and fixed with interference screws, creating two fixation points that control scaphoid pronation while permitting early motion without Kirschner wires; in 27 chronic cases, grip, pain, and DASH improved, though the radiographic gap often persisted [6]. Carratalá et al. positioned a graft along the SL rotational axis with dorsal reinforcement under dry arthroscopy in nine reducible injuries, preserving the dorsal secondary stabilizers [22]. More recently, all-dorsal arthroscopic ligamentoplasty (ADAL) combined a palmaris longus graft, a FiberTape internal brace anchored with a SwiveLock, and dorsal capsulodesis, avoiding palmar approaches and complete transosseous tunnels [14]; in twelve patients with stage 4–5 instability, Ribolzi and Merlini reported the visual analog scale falling from 6 to 1, a flexion–extension arc of 144°, grip near 180% of baseline, and improved radiographic alignment, noting that restoring radioscaphoid congruence may matter more than fully closing the gap [15].

At the highest grades, volar stabilization procedures may be added to complement the dorsal repair: arthroscopic imbrication of the long radiolunate ligament (MIRLIN) as an adjunct [25], and an open volar scaphotrapeziotrapezoid reconstruction to control scaphoid rotation and augment the dorsal repair [24]. These techniques have been reported to relieve pain and improve grip and motion, but the series are small, short-term, and uncontrolled, residual diastasis is common, and no single approach has been shown to be superior in comparative studies [16,29].

### 3.6. A Proposed Stage- and Ligament-Based Conceptual Framework for Arthroscopic Treatment Selection

**Figure 3:**
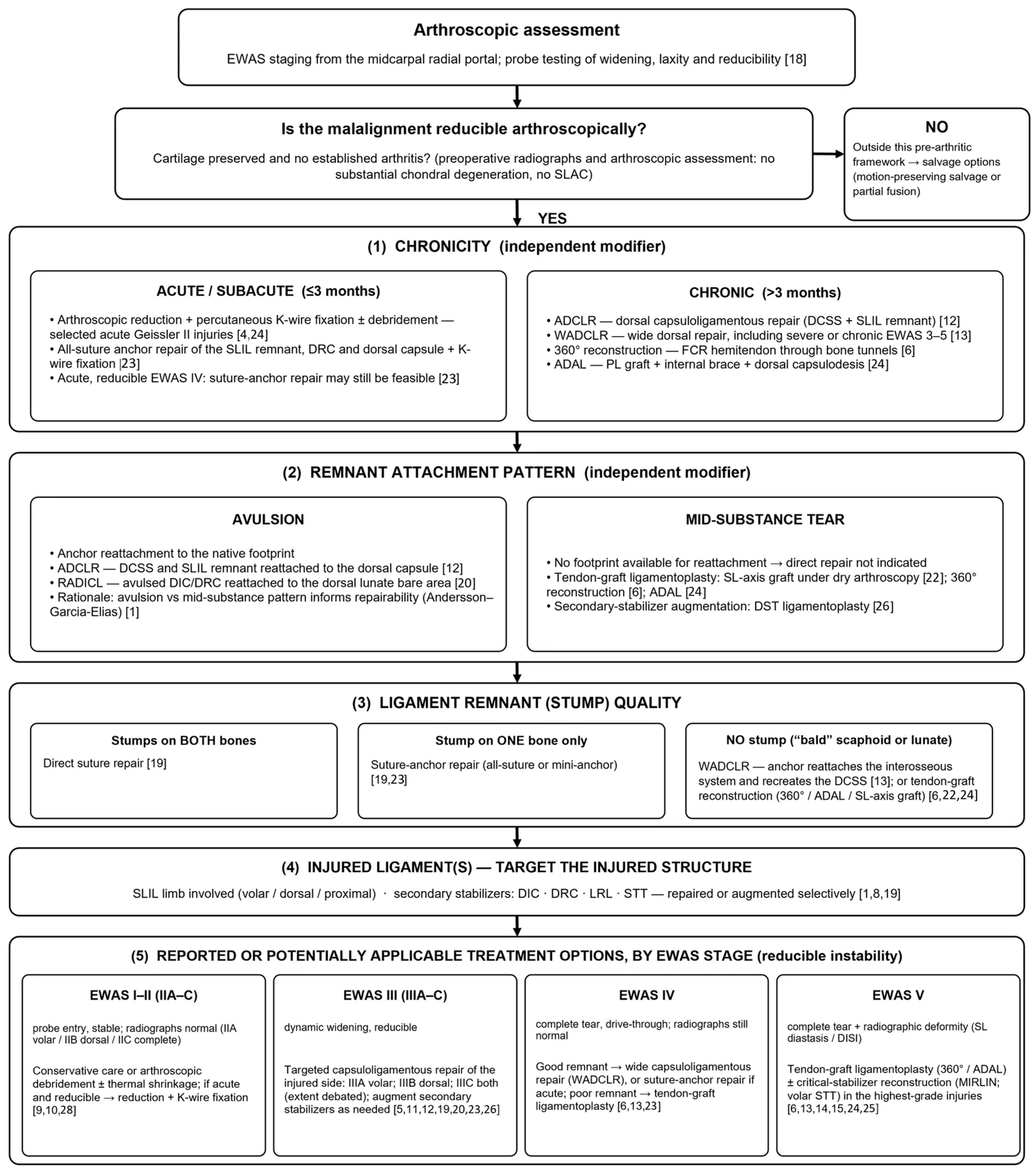
Proposed decision flow for arthroscopic treatment selection in pre-arthritic scapholunate instability. After arthroscopic assessment with EWAS staging, the pathway first confirms that the malalignment is reducible and that cartilage is preserved without established arthritis, integrating the preoperative radiographs with the arthroscopic findings. Treatment is then refined sequentially by injury chronicity (acute/subacute versus chronic), the attachment pattern of the ligament remnant (avulsion versus mid-substance), remnant quality (stumps on both bones, on one bone only, or absent), and the specific intrinsic and extrinsic ligament(s) involved, leading to the reported or potentially applicable treatment options by EWAS stage. Treatment choice is further modified at every step by cartilage status, radiographic deformity, and surgeon experience. Irreducible deformity, substantial cartilage loss, and established scapholunate advanced collapse fall outside this pre-arthritic pathway and require consideration of a different treatment pathway, including salvage procedures where appropriate. This is a pragmatic, opinion-based framework and not a validated clinical guideline. The framework synthesizes small case series (mostly Level IV) and technique reports; no arthroscopic technique has been shown to be superior to alternative operative or nonoperative approaches in comparative clinical studies, and the certainty of the overall evidence is low [16,29]. ADCLR, arthroscopic dorsal capsuloligamentous repair; WADCLR, wide ADCLR; ADAL, all-dorsal arthroscopic ligamentoplasty; RADICL, repair/augmentation of dorsal intercarpal ligament; DCSS, dorsal capsulo-scapholunate septum; DST, dorsal scaphotriquetral; MIRLIN, minimally invasive radiolunate imbrication neutralization; STT, scaphotrapeziotrapezoid; DIC, dorsal intercarpal ligament; DRC, dorsal radiocarpal ligament; LRL, long radiolunate ligament; SLIL, scapholunate interosseous ligament; PL, palmaris longus; FCR, flexor carpi radialis; DISI, dorsal intercalated segment instability; SLAC, scapholunate advanced collapse. Arrows between boxes indicate the direction of the decision pathway, and the branch to the right denotes injuries that fall outside this pre-arthritic framework; within the boxes, an arrow (→) should be read as ‘leads to’. The treatment options and concepts shown in the figure are described in references [1,5,6,8,9,10,11,12,13,14,15,18,19,20,22,23,24,25,26,28].

Because arthroscopy simultaneously establishes the diagnosis, grading instability, confirming reducibility and cartilage status, and gauging ligament repairability, it may help to guide treatment. On this basis we propose a pragmatic, stage- and ligament-based conceptual framework that relates each intervention to the EWAS/Geissler grade and to the specific intrinsic and extrinsic ligaments injured (Table 3, Figure 3) [2,8]. This framework is opinion-based and derived largely from small case series and technique reports; it has not been clinically validated and is intended to organize the available options rather than to serve as an evidence-based guideline. Within any given stage, the choice among debridement, repair, capsulodesis, and reconstruction is further governed by injury chronicity (acute, subacute, or chronic), the quality and attachment pattern of the ligament remnant (avulsion versus mid-substance; stumps present on both bones, on one bone only, or absent), reducibility of the malalignment, cartilage status, and the presence or absence of radiographic deformity. Reducibility and the absence of arthritis are confirmed arthroscopically before committing to repair versus reconstruction (Figure 3).

Occult and partial, stable tears (EWAS I–II) are managed conservatively or with arthroscopic debridement and electrothermal shrinkage, with reduction and pinning reserved for an acute, reducible lesion [9,10,28]. Partial-to-complete reducible tears (EWAS III) are treated by capsuloligamentous repair targeted to the injured limb, volar (IIIA) or dorsal (IIIB), and for complete reducible tears (IIIC), by dorsal repair with or without an added volar repair, an extent that remains debated; deficient secondary stabilizers are augmented as needed [5,11,12,19,20,23,26]. For complete, reducible injuries with or without radiographic deformity (EWAS IV–V), wide capsuloligamentous repair or tendon-graft ligamentoplasty is selected according to the quality of the remaining ligament, with critical-stabilizer reconstruction added at the highest grades [6,13,14,15,24,25]. Throughout, the quality of the ligament remnant, whether satisfactory stumps are present on both bones, on one bone only, or absent, guides the choice among suture repair, suture-anchor repair, and reconstruction [19].

For the purposes of this framework, “arthroscopic” denotes procedures performed entirely under arthroscopic control; “arthroscopic-assisted” denotes procedures in which arthroscopy is used to guide or facilitate the principal procedure, while percutaneous or limited accessory approaches are used for fixation, graft passage, or other essential steps; and “hybrid” denotes procedures combining an arthroscopic component with a formal open component that contributes materially to stabilization, such as arthroscopic dorsal repair combined with open volar STT reconstruction [22]. Irreducible deformity, substantial cartilage degeneration, and established SLAC fall outside this pre-arthritic, reducible-instability framework and require consideration of a different treatment pathway, including salvage procedures where appropriate.

Postoperative rehabilitation also differs substantially among these techniques. After ADCLR, the wrist is immobilized in extension for six weeks after suture repair alone and for eight weeks when K-wires are added [12]; after RADICL, by contrast, hand therapy begins on the first postoperative day with protected inner-range motion, strengthening from 10 to 12 weeks, and a target return to full activity by 16 weeks [20]; and after ADAL, the wrist is splinted in 30 degrees of extension for four weeks before supervised therapy begins, with rehabilitation continuing for approximately three months and heavy loading avoided until three months [15]. Immobilization duration, temporary pinning and the timing of wire removal, and the progression of motion and loading are therefore heterogeneous across series. This heterogeneity is an important potential source of variation when outcomes are compared across techniques.

Despite the coherence of this framework, the optimal choice remains uncertain: a recent scoping review found no clearly superior technique, and a meta-analysis judged the overall evidence for wrist arthroscopy to be of low certainty [16,29]. Selection therefore still rests on arthroscopic staging, chronicity, and surgeon experience.

## 4. Summary of Findings

Wrist arthroscopy now serves as both a reference standard for diagnosis and grading and the platform for stage-based, ligament-targeted treatment, with the dry technique enabling hybrid arthroscopic–mini-open procedures [17,18,21]. For occult and partial tears (EWAS I–II), debridement, electrothermal shrinkage, and reduction–pinning have been reported to relieve pain and preserve motion in most patients, and a systematic review of predominantly Level IV case series described improvements in pain, range of motion, grip, and the radiographic gap; because its untreated cohort was drawn from a separate study rather than a concurrent control group, that comparison is indirect and does not establish efficacy relative to no treatment [4,9,10,27,28]. For partial-to-complete reducible tears (EWAS III), dorsal, volar, or combined capsuloligamentous repair, with selective augmentation of the DIC/DRC, scaphotriquetral, or long radiolunate ligaments, has been associated with favorable short-term functional outcomes [11,12,19,20,23,26]. For advanced reducible injuries (EWAS IV–V), wide repair and tendon-graft ligamentoplasty have been associated with improvements in pain, grip, motion, and radiographic alignment in small observational series [6,13,14,15,22,24].

The potential advantages of arthroscopic treatment, namely possible preservation of the secondary stabilizers and potentially less postoperative stiffness, are described across techniques and stages; direct comparative evidence against open surgery is lacking, so comparative superiority cannot be established. The proposed preservation of proprioception is an anatomical or theoretical inference based on preservation of capsular and neural structures rather than a clinically demonstrated postoperative outcome, as none of the studies cited here directly measured postoperative proprioception. Preservation of capsular vascularity is likewise inferred from the anatomy spared at surgery, and no direct comparative clinical evidence establishes either benefit [1,3]. However, the findings are not uniform: radiographic diastasis frequently persists despite clinical improvement, higher-grade injuries are less predictable, and conventional repair may be insufficient for advanced instability, as reflected in the 19% residual DISI after dorsal repair [6,12,13]. Reported complications include complex regional pain syndrome and hardware migration; after transosseous reconstruction, scaphoid tunnel screw migration required screw removal in two patients [6]. Observed complications should be distinguished from potential risks: tunnel breakage or fracture and scaphoid or lunate avascular necrosis are described as theoretical risks of drilling transosseous tunnels through these small bones rather than as events observed in that series [6]. Synthetic-tape and suture-anchor augmentation, by contrast, may be associated with implant-related complications: Lee and Shin reported progressive carpal osteolysis and fragmentation in three patients after synthetic tape–augmented scapholunate reconstruction. All three constructs used synthetic tape secured with SwiveLock suture anchors; in one case, the tendon–tape construct was additionally passed through a bone tunnel and secured with two interference screws. Two of the three patients required salvage procedures. Importantly, these cases were not performed as SL 360°, Anatomic Front and Back (ANAFAB), or all-dorsal internal-brace reconstructions; the original report explicitly noted that osteolysis or bony fragmentation had not been reported for those techniques at one to three years of follow-up. Because only three cases have been reported, these observations should be regarded as construct-specific and should not be generalized to synthetic tape- or anchor-augmented reconstruction as a whole [30]. The recent shift toward anchor- and tape-based, tunnel-sparing constructs therefore avoids tunnel-related fracture but does not eliminate implant-related morbidity, underscoring the need for cautious patient selection and close radiographic follow-up [13,14,30].

Taken together, the adverse events reported across the series in Table 2 span stiffness and loss of motion; recurrent instability or recurrent diastasis (for example, 1 of 19 wrists after suture-anchor repair [23]); persistent DISI (43 of 221 after dorsal capsuloligamentous repair [12]); complex regional pain syndrome; pin- and hardware-related problems, including screw migration requiring removal in two patients after transosseous reconstruction [6]; implant-related osteolysis [30]; and conversion to salvage surgery. Tunnel fracture and avascular necrosis are considered separately as potential risks rather than observed complications, since the transosseous reconstruction series reported them as theoretical risks and recorded neither event in its patients [6]. Denominators and follow-up periods, where reported, are given in Table 2; many series, however, report complications incompletely, and follow-up is generally too short to capture late degeneration. Importantly, short-term symptomatic improvement should be distinguished from long-term disease modification: patient-reported outcomes frequently improve despite persistent SL widening or DISI, and there is currently little evidence that arthroscopic procedures reliably restore normal carpal mechanics or prevent progression to SLAC [16,27,29].

Crucially, the evidence base is dominated by small, retrospective Level III–IV case series; the available systematic reviews emphasize marked heterogeneity and identify no clearly superior technique [27,29]. Only one systematic review and meta-analysis of randomized trials addresses wrist arthroscopy directly, and it judged the benefit over open or non-surgical treatment to rest on low-certainty evidence [16]. High-level, arthroscopy-specific comparative data therefore remain scarce, and current conclusions should be regarded as provisional.

## 5. Limitations

This is a narrative, non-systematic review. The literature search was selective and purposive, and sources were chosen for their relevance to the review question, their methodological or technical contribution, and their clinical importance, without a registered protocol, formal risk-of-bias appraisal, or meta-analysis. This purposive selection process may introduce selection bias. The primary studies cited here are predominantly small, single-center, retrospective Level III–IV case series with short follow-up, and several newer techniques are reported only by their originating centers, raising expertise and publication bias. Indeed, much of the available literature originates from a relatively small number of pioneering surgeons and specialist institutions. This concentration of evidence may reflect the specialised nature and relatively recent development of these techniques. Their results reflect exceptional experience with technically demanding procedures, and advanced arthroscopic repair and reconstruction carry a substantial learning curve, so outcomes reported by these centres may not be reproducible in general practice, an effect that has not been formally studied. Several of the newest techniques, including those incorporated into the proposed framework, have not yet undergone independent external validation, and their early results should be interpreted in light of this limitation. Heterogeneous classifications (Geissler, EWAS, Garcia-Elias), inconsistent indications, and varied outcome measures preclude direct comparison, and the available systematic reviews largely pool open and arthroscopic procedures rather than isolating arthroscopic outcomes [16,27,29]. The evidence is also imbalanced, being rich in technical description but sparse in high-level therapeutic data. Future work should adopt a standardized, arthroscopy-based classification and a core outcome set; conduct prospective, stage-stratified comparative or randomized trials with longer follow-up; and specifically determine whether arthroscopic repair and reconstruction maintain carpal alignment, prevent progression to scapholunate advanced collapse, and justify their cost and learning curve relative to open techniques.

## 6. Conclusions

Arthroscopy allows for direct assessment of the scapholunate interval, and a stage- and ligament-based approach may preserve the secondary stabilizers and native capsuloligamentous structures and may reduce the stiffness associated with open surgery. Improvements in pain, grip strength, and motion have been reported after arthroscopic treatment, but almost entirely in small, heterogeneous, uncontrolled series and technique reports with short follow-up. Radiographic alignment is not always restored or maintained, and higher-grade injuries remain less predictable. The stage- and ligament-based framework proposed here is therefore a conceptual, opinion-based construct that has not been clinically validated; it is intended to organize current options and to structure future study rather than to serve as a treatment guideline. No arthroscopic technique has been shown to be superior to alternative operative or nonoperative approaches in comparative clinical studies, and whether these procedures restore normal carpal mechanics or prevent progression to scapholunate advanced collapse remains unproven. In practice, the framework applies to early, reducible injuries without arthritis. These procedures require substantial arthroscopic expertise and may not be readily generalisable beyond experienced wrist arthroscopy centres. Prospective, stage-stratified comparative studies with standardized classification, a core outcome set, long-term follow-up, and independent external validation are required before the relative merits of these techniques can be established.

## Figures and Tables

**Table 1:** Comparison of the Geissler (1996) [17] and EWAS (2013) [18] arthroscopic classifications of scapholunate instability.

Geissler 1996 (Finding) [17]	EWAS 2013 (Finding) [18]	Key Difference
I: interosseous ligament attenuation/hemorrhage from the radiocarpal space; no carpal incongruency in the midcarpal space	I: no passage of the probe (synovitis; stable)	Mildest, stable partial injury
II: attenuation/hemorrhage from the radiocarpal space with scaphoid–lunate incongruency/step-off in the midcarpal space; slight gap (<probe width)	II: probe passes into the SL space without widening (stable). In the expanded form used in subsequent clinical practice this stage is subdivided as IIA volar, IIB dorsal, and IIC complete passage without widening	Partial, stable, but EWAS additionally records the site and extent of the lesion
III: incongruency in both radiocarpal and midcarpal spaces; probe passes through the gap	IIIA/IIIB/IIIC: dynamic widening, subdivided as volar/dorsal/complete	EWAS subdivides by tear location
IV: gross instability; a 2.7 mm arthroscope passes (“drive-through”)	IV: arthroscope passes midcarpal → radiocarpal, radiographs normal	EWAS additionally requires normal radiographs
Not applicable (Geissler ends at grade IV; no grade V)	V: arthroscope passes with radiographic deformity (SL diastasis, DISI)	Only EWAS grades radiographic deformity (reconstruction/salvage)

Note: The EWAS and Geissler classifications are not directly equivalent; the EWAS system additionally incorporates the anatomical location and extent of the SLIL injury, so the correspondence shown here is approximate and not a standardized 1:1 mapping. The stage II subdivisions (IIA–IIC) are not part of the original 2013 description and derive from the expanded form of the classification used subsequently in clinical practice [12]. EWAS, European Wrist Arthroscopy Society; DISI, dorsal intercalated segment instability; SL, scapholunate. The arrow (→) indicates the direction of arthroscope passage, from the midcarpal to the radiocarpal joint.

**Table 2:** Reported arthroscopic and arthroscopic-assisted techniques for scapholunate instability, by study design and level of evidence (chronological).

Author (Year)	Study Design (LoE)	*n*; Acute/Chronic	Target & Technique	Follow-Up	Key Outcomes (pre → post)	Complications/Failures
Weiss (1997) [9]	Retrospective case series (IV)	*n* = 43 wrists; mostly partial, chronic (SL/LT tears)	Partial/complete SLIL; arthroscopic debridement alone	Mean 27 mo	Symptom relief in ~85% of partial vs. ~66% of complete tears; grip +23%; no progression to static instability	None reported
Darlis (2005) [10]	Retrospective case series (IV)	*n* = 16; partial, chronic	Partial SLIL (membranous/volar); debridement + bipolar RF thermal shrinkage	Mean 19 mo	14/16 substantial pain relief (8 pain-free); F–E arc 142°; radiographic stability maintained	None
del Piñal (2011) [11]	Technique description (no clinical series)	4 preliminary cases (insufficient follow-up); volar SLIL laxity	Volar SLIL (+ volar capsule/LRL); all-inside arthroscopic volar SL suture	NR	Closes anterior SL gap; avoids a volar open approach	NR (technique)
Bednar (2015) [4]	Narrative/technique review (not primary data)	N/A	SLIL tear; debridement ± thermal shrinkage; acute reduction + K-wire/screw	N/A	Summarizes pain relief for partial tears; acute reduction–pinning success if <3 mo	N/A (review)
Mathoulin (2016) [12]	Retrospective case series (IV)	*n* = 221; chronic, dynamic	DCSS + dorsal SLIL remnant; ADCLR ± pinning	Mean 39.4 mo	Satisfaction in 215/221 (reported as 95.7%); DASH 47.04 → 9.4; extension +14°; grip 93.4% of contralateral	Residual DISI 43/221 (19%); 5 fair, 1 poor (failures were Garcia-Elias V)
Carratalá (2016) [22]	Technique report + early series (Arthrosc Tech)	*n* = 9; chronicity NR; dynamic/easily reducible	SL axis + dorsal; arthroscopically assisted ligamentoplasty (PL/FCR graft + screw/anchor; dry)	Early (short-term)	Favorable early results; soft-tissue/PIN preservation	Perioperative fracture and diastasis recurrence described as potential risks, not reported as observed events; rates NR
Corella (2017) [6]	Case series within a technique article (IV)	*n* = 27 (20M/7F); chronic	SL volar + dorsal; arthroscopic 360° reconstruction (FCR hemitendon, tunnels, interference screws)	Serial, 3 mo to ≥2 y (*n* = 9–11 at ≥2 y)	VAS 5 → 0.4; DASH 35.3 → 4.2; grip 33 → 46 kg (at ≥2 y)	Scaphoid tunnel screw migration requiring removal (n = 2); volar tunnel pain; residual SL gap in some. Tunnel fracture and scaphoid/lunate AVN reported as theoretical risks, not observed
Williams (2022) [20]	Book chapter; embedded institutional case series	*n* = 5 (3F/2M); indications not separately specified	DIC + DRC dorsal-lunate reinsertion (RADICL)	6 mo	Pain 49.4 → 23.8; PRWE 65.6 → 27.3; QuickDASH 50.9 → 27.7; grip 19.6 → 33.6 kg	NR
de Villeneuve Bargemon (2023) [13]	Prospective single-centre series (IV)	*n* = 112; EWAS 3–5, incl. severe/chronic	Dorsal capsuloligamentous complex; WADCLR (± anchor)	3–12 mo	Radiographic parameters normalized; function improved except flexion	Few; 1 complication reported
Lee (2023) [23]	Retrospective case series (IV)	*n* = 19; acute/subacute (<3 mo), Geissler III	SLIL remnant + DRC + dorsal capsule; mini all-suture anchor + K-wire	Mean 26.5 mo	18/19 (94.7%) good/excellent MMWS; grip +14.7%	1 recurrence
Smith (2024) [24]	Case reports (n = 2)	*n* = 2; EWAS V (reducible, radiographic deformity)	Volar STT reconstruction after arthroscopic ADCLR (hybrid)	12 mo	VAS 0; excellent QuickDASH; full return to manual work	None reported
Kakar (2025) [25]	Technique description (illustrative cases)	NR (illustrative); reducible	LRL imbrication (MIRLIN; adjunct to SLIL repair/RADICL)	Case examples (3.5–6 mo)	Resolution of SL gap/DISI and improved Mayo/PRWE in reported cases	NR
Chambers (2026) [19]	Retrospective single-centre case series (IV)	*n* = 8; predynamic, EWAS IIIA–C (no DISI)	Dorsal ± volar capsuloligamentous shuttling; modified Nice knot	Mean 18.9 mo	Pain relief; excellent subjective function and ROM	None reported
Im (2026) [26]	Retrospective case series (IV)	*n* = 13 isolated SL; dynamic, Geissler II–III	Dorsal scaphotriquetral (DST) ligamentoplasty (SutureTape + PushLock)	To 12 mo	VAS 7.9 ± 1.5 → 1.4 ± 2.4; grip 27.4 ± 10.7 → 37.3 ± 10.2 kg; modified Mayo 46.5 ± 12.9 → 89 ± 11.0	No major complications
Ribolzi & Merlini (2026) [15]	Prospective case series (IV)	*n* = 12; chronic, reducible	Dorsal SL complex (SLIL + DIC); ADAL: PL graft + FiberTape internal brace + dorsal capsulodesis	Mean 15.9 mo	VAS 6 → 1; F–E 144°; grip ~180% of preop; Mayo 79.5; radiographic improvement	None observed

LoE, level of evidence (Oxford CEBM); NR, not reported; N/A, not applicable; mo, months; y, years. Outcome values are presented in the preoperative → postoperative direction and are given with measures of variability (mean ± SD or range) where the original study reported them; values that were not reported are marked NR and have not been estimated or inferred. Sample size refers to patients or wrists as specified by the original study. For patient-reported outcome measures, lower scores indicate better function or fewer symptoms; for grip strength and range-of-motion measures, higher values generally indicate improvement. Radiographic measures are reported according to the direction described in the original study. Study types are heterogeneous and should not be interpreted as providing equivalent levels of clinical evidence. Technique descriptions without a clinical series, case reports, book-chapter-embedded case series, and narrative reviews are presented for technical or contextual relevance and are not equivalent to comparative or larger clinical series. The RADICL clinical data (Williams (2022) [20]) are an institutional case series (n = 5, 6-month follow-up) reported within a book chapter rather than a separate peer-reviewed study; indications for the individual patients are not separately specified in that source. Mathoulin (2016) [12] reported satisfaction in 215 of 221 patients (reported as 95.7%); the stated percentage does not correspond arithmetically to the reported denominator, and the published value has been retained without recalculation. SLIL, SL interosseous ligament; DIC, dorsal intercarpal; DRC, dorsal radiocarpal; DCSS, dorsal capsulo-scapholunate septum; DST, dorsal scaphotriquetral; LRL, long radiolunate; STT, scaphotrapeziotrapezoid; ADCLR, arthroscopic dorsal capsuloligamentous repair; WADCLR, wide ADCLR; ADAL, all-dorsal arthroscopic ligamentoplasty; RADICL, repair/augmentation of dorsal intercarpal ligament; MIRLIN, minimally invasive radiolunate imbrication neutralization; PL, palmaris longus; FCR, flexor carpi radialis; RF, radiofrequency; VAS, visual analog scale; DASH, Disabilities of the Arm, Shoulder and Hand; QuickDASH, shortened DASH; PRWE, Patient-Rated Wrist Evaluation; MMWS/Mayo, Modified Mayo Wrist Score; F–E, flexion–extension; PIN, posterior interosseous nerve; AVN, avascular necrosis.

**Table 3:** Proposed pragmatic, stage- and ligament-based conceptual framework for arthroscopic treatment selection in pre-arthritic scapholunate instability (an opinion-based framework, not a validated clinical guideline).

EWAS Stage (Approximate Geissler Correspondence)	Key Arthroscopic Finding	Reported or Potentially Applicable Treatment Options	Refs.
I (I)	SLIL hemorrhage/attenuation; probe cannot pass; stable	Conservative (proprioceptive rehabilitation); debridement if symptomatic	[9]
II (IIA–C)	Midcarpal step-off; probe entry without dynamic widening; stable (IIA volar, IIB dorsal, IIC complete passage)	Debridement ± electrothermal shrinkage; ± reduction and pinning if acute	[9,10,28]
IIIA	Volar widening (anterior laxity)	Volar capsuloligamentous repair (all-inside)	[11,19]
IIIB	Dorsal widening (posterior laxity)	Dorsal capsuloligamentous repair (ADCLR) ± extrinsic-stabilizer restoration (RADICL; dorsal scaphotriquetral ligamentoplasty)	[12,20,26]
IIIC	Complete but reducible (volar + dorsal)	Dorsal ± added volar capsuloligamentous repair (combined vs. dorsal-focused debated), or suture-anchor repair + dorsal capsulodesis; reconstruction if remnant insufficient	[5,6,19,23]
IV	Drive-through (complete); reducible; normal radiograph	WADCLR or tendon-graft ligamentoplasty; suture-anchor repair if acute	[6,13,23]
V	Reducible with radiographic deformity (gap/DISI)	Tendon-graft ligamentoplasty (360°/ADAL) ± critical-stabilizer reconstruction (MIRLIN; volar STT) ± CRPP	[6,14,15,24,25]

This table is a pragmatic, opinion-based conceptual framework, not a validated clinical guideline; most listed options are supported only by small case series or technique reports. See Table 2 for the study design, level of evidence, and reported complications of the supporting literature. Treatment within each stage is further modified by chronicity, ligament quality and attachment pattern (avulsion vs. mid-substance; remnant on both bones, one bone, or absent), reducibility, cartilage status, and radiographic deformity (Figure 3). Irreducible deformity, substantial cartilage loss, and established scapholunate advanced collapse (SLAC) fall outside this pre-arthritic framework and require consideration of a different treatment pathway, including salvage procedures where appropriate. ADCLR, arthroscopic dorsal capsuloligamentous repair; WADCLR, wide ADCLR; ADAL, all-dorsal arthroscopic ligamentoplasty; STT, scaphotrapeziotrapezoid; SLIL, SL interosseous ligament; DISI, dorsal intercalated segment instability.

## Data Availability

No new data were created or analyzed in this study. Data sharing is not applicable to this article.
